# Simultaneous high‐pitch multi‐energy CT pulmonary angiography using a dual‐source photon‐counting‐detector CT: A phantom experiment

**DOI:** 10.1002/acm2.14496

**Published:** 2024-08-29

**Authors:** Jelena M. Mihailovic, Michael R. Bruesewitz, Joseph R. Swicklik, Mariana Yalon, Prabhakar S. Rajiah, Joel G. Fletcher, Cynthia H. McCollough, Lifeng Yu

**Affiliations:** ^1^ Department of Radiology Mayo Clinic Rochester Minnesota USA

**Keywords:** dual source computed tomography, photon‐counting‐detector computed tomography, pulmonary embolism

## Abstract

**Purpose:**

A dual‐source CT system can be operated in a high‐pitch helical mode to provide a temporal resolution of 66 ms, which reduces motion artifacts in CT pulmonary angiography (CTPA). It can also be operated in a multi‐energy (ME) mode to provide iodine maps, beneficial in the evaluation of pulmonary embolism (PE). No energy‐integrating detector (EID) CT can perform simultaneous ME and high‐pitch acquisition. This phantom study aimed to evaluate the ability of a photon‐counting‐detector (PCD) CT to perform simultaneous high‐pitch and ME imaging for CTPA.

**Methods:**

A motion phantom was used to mimic the respiratory motion. Two tubes filled with iodine with intravascular thrombus mimicked by injecting glue within the tubes were placed along with 5, 10, and 15 mg/mL iodine samples, on a motion phantom at 20 and 30 revolutions per minute. Separate high‐pitch and ME EID‐CT scans and a single high‐pitch ME PCD scan were acquired and virtual monoenergetic images and iodine maps reconstructed. Percent thrombus occlusion was measured and compared between static and moving images.

**Results:**

When there was motion, EID‐CT ME suffered from significant motion artifacts. The measured iodine concentrations with PCD‐CT in high‐pitch ME were more stable when there was a motion, with a lower standard deviation than EID‐CT in ME mode. The estimated percent thrombus occlusion dropped significantly with applied motion on EID‐CT, while PCD‐CT high‐pitch ME mode showed good agreement between measurements on static or moving images.

**Conclusion:**

PCD‐CT with combined ME and high‐pitch mode facilitates simultaneous accurate iodine quantification and assessment of intravascular occlusion.

## 1 INTRODUCTION

Pulmonary embolism (PE) is a common and often lethal complication of venous thromboembolic disease.^1^ When a thrombus partially or entirely blocks a pulmonary artery, the affected region of the lung is ventilated but not perfused. A timely diagnosis of PE is crucial to implement treatment accurately and improve patient outcomes. Multiple imaging modalities are available to evaluate PE: computed tomography pulmonary angiography (CTPA),^2^ magnetic resonance pulmonary angiography,^3^, ^4^ and nuclear medicine ventilation/perfusion scans.^5^ Each imaging modality is unique concerning its advantages and limitations, diagnostic performance, invasiveness, local availability and expertise, spatial and temporal resolution, and presence of the motion artifacts.^6^

However, compared with traditional gold‐standard invasive pulmonary angiography, CTPA has become the first‐line imaging modality with high sensitivity and specificity for the workup of patients with suspected PE. Studies have shown that CTPA can be used as a single diagnostic test to rule out or establish acute PE.^7^, ^8^, ^9^, ^10^, ^11^ The presence of motion artifacts, especially on older scanners, can result in misinterpretation or reduce the ability to detect an embolus.

Advances in CT technology have enabled CTPA acquisition with lower radiation doses and volumes of iodinated contrast agents.^2^, ^12^ Adopting dual‐source CT scanners in routine practice makes it possible to perform scans in a high‐pitch helical mode (“FLASH” mode) with a temporal resolution of up to 66 ms and table travel as fast as 737 mm/s, reducing motion artifacts.^13^ A dual‐source CT can also be operated in a multi‐energy (ME) mode to provide pulmonary perfused blood volume information, which is beneficial in the evaluation of PE. ME can generate iodine maps, allowing visualization of the iodine distribution within lungs, so quantitative volume or region of interest (ROI) analysis can be performed,^14^ and virtual monoenergetic images (VMIs), with low energy VMIs used to increase intravascular iodine signal.^15^ It has been shown that iodine maps can help to depict the effects of acute and chronic PE on parenchymal perfusion, leading to improved detection of small, occluding emboli and estimation of the extent of pulmonary perfusion defects.^16^ The iodine concentration in the lung has been demonstrated to be an effective surrogate for pulmonary blood flow that can only be acquired using dynamic imaging methods.^17^

However, the existing dual‐source CT based on energy‐integrating detectors (EIDs) does not allow simultaneous ME acquisition at a high temporal resolution in a high‐pitch mode. The recently introduced clinical dual‐source photon‐counting‐detector (PCD)‐CT system has an inherent ME imaging capability.^18^, ^19^ Because of the multi‐energy imaging capability for each of the two x‐ray tube‐detector systems, the dual‐source PCD‐CT can perform ME imaging during the high‐pitch scan. By simultaneously combining the high‐pitch scan mode with ME acquisition, PCD‐CT can achieve the highest temporal resolution and shortest scan time, reduced motion artifact, and more accurate quantitative iodine measurements from ME imaging.

The purpose of this study is to use a dynamic phantom to evaluate the ability of a clinical dual‐source PCD‐CT to perform simultaneous ME and high‐pitch imaging for CTPA.

## 2 METHODS

**FIGURE 1:**
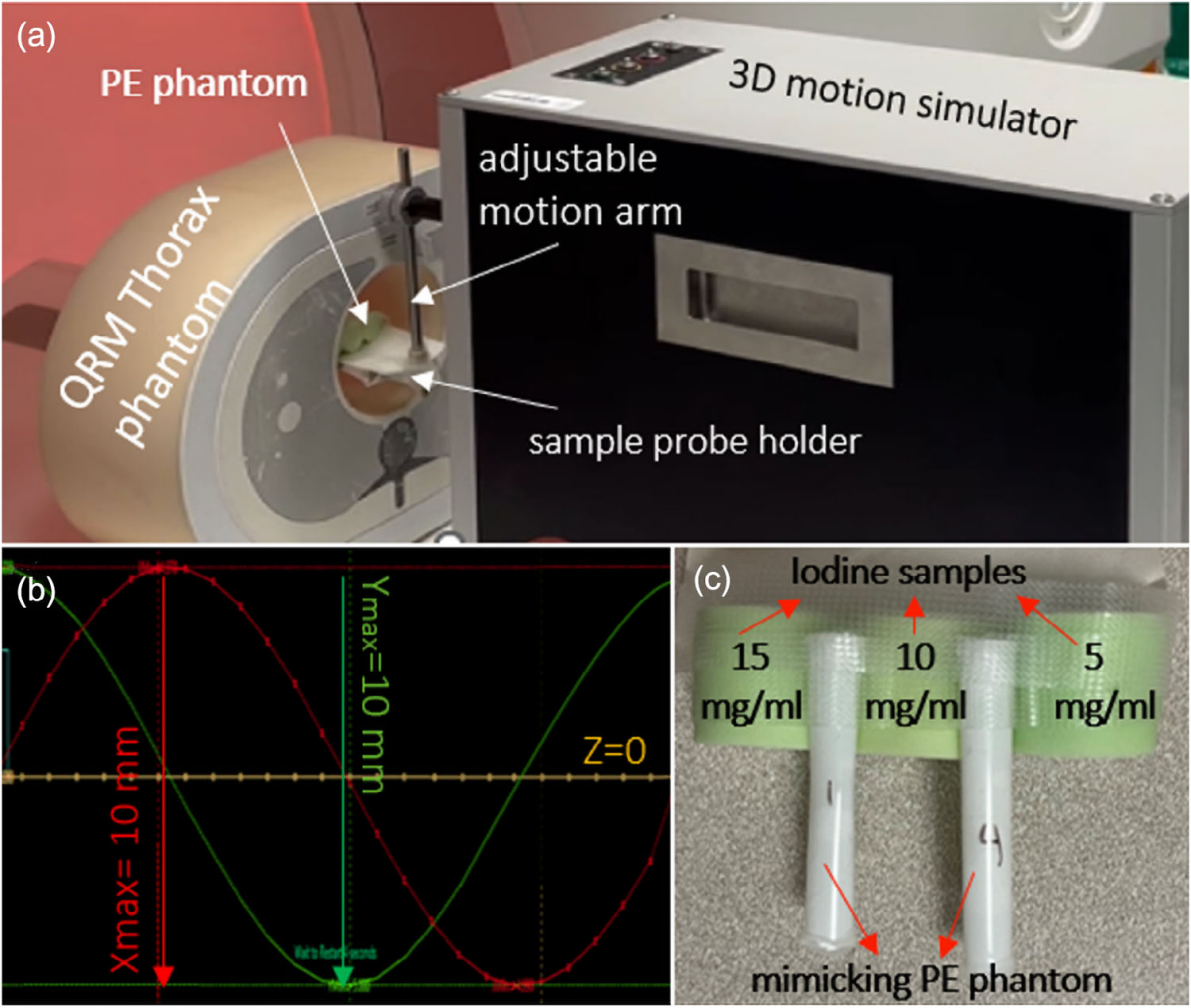
Experimental set up. (a) The QRM‐Sim4D with QRM Thorax phantom and PE phantom. (b) Projection of the X‐Y circular motion pattern. (c) Mimicking PE phantom with three different concentration iodine filled tubes. PE, pulmonary embolism.

In this study, a motion phantom was used to mimic the potential motion caused by respiration in the lung (QRM‐Sim4D‐Cardio; Quality Assurance in Radiology and Medicine, Möhrendorf, Germany). The phantom consists of a motion arm controlled by a motor and a computer (Figure 1a). Motion patterns of the arm were programmed on a computer connected to the motion arm. Two motion patterns were programmed with a circular trajectory in the scanner axial, X‐Y plane (magnitude 10 mm) with a motion frequency of 20 revolutions per minute (rpm) and 30 rpm (Figure 1b).

Parameters were selected based on the studies by Tamiya^20^ and Tashiro^21^ who found chest wall displacement due to respiration motion is the biggest in ventral‐dorsal and left‐right up to 10 mm. Two plastic tubes (diameter, 4.5 mm) were built to mimic two parallelly positioned vessels (Figure 1c). Hot glue was used to simulate intravascular thrombus, which was hemispherical tin shape. In the first tube, the percentage of intravascular thrombus occlusion was kept at less than ∼75%, while in the second, the percentage of intravascular occlusion was more than ∼75%. We found that the hot glue is an excellent substitute to mimic PE or intravascular occlusion due to its primary material, ethylene vinyl acetate.^22^ The tubes were filled with an iodine solution (Omnipaque 350 mg I/mL; GE HealthCare). The concentration of iodine mixture in tubes was 15.5 mg/mL. Three circular solid iodine samples at various concentrations (5, 10, and 15 mg/mL) (Sun Nuclear Corp., Melbourne, Florida, USA) were placed beneath the two vessel tubes. They were attached to the motion arm to simulate the motion of vessels during breathing (20 and 30 rpm) (Figure 1c). The range of iodine concentrations of circular phantoms was determined to cover the in vivo iodine density range.

The motion arm, along with the attached vessel phantom, was placed inside a semi‐anthropomorphic chest phantom (Thorax‐CCI, QRM Inc., Möhrendorf, Germany) at a dimension of 25 × 35 cm and scanned using both a clinical dual‐source EID CT (Somatom Force, Siemens Healthineers, Forchheim, Germany) and a dual‐source PCD‐CT (Naeotom Alpha, Siemens Healthineers) system without and with motion of the vascular PE phantom. Each scan was repeated twice.

EID‐CT scans were performed separately using the high‐pitch or ME mode. The tube potential was 120 kV for the high‐pitch mode, and the quality reference mAs was 84. The volume CT dose index (CTDIvol) was 5.48 mGy. The tube potentials for the EID‐CT ME mode were 90 and 150 with an added tin filter (Sn150) for tubes A and B, respectively. The corresponding quality reference mAs were 105 and 81, respectively, with a pitch of 0.6. CTDIvol was 5.37 mGy. The detector collimation was 96 × 0.6 mm with a z‐flying focal spot for both scan modes. Images were reconstructed at a slice thickness of 1.5 mm with an increment of 1 mm using a medium‐sharp body kernel, Br44 with an iterative reconstruction strength setting of 3 (ADMIRE, Siemens Healthineers). In addition, EID‐CT ME images were reconstructed using a smooth quantitative kernel, Qr44‐3, at different VMI energies of 50, 60, and 70 keV.

Dual‐source PCD‐CT was performed using the high‐pitch ME mode at 120 kV. Automatic exposure control was used with a CAREkeV IQ level of 150. The helical pitch was 3.2 to achieve the best temporal resolution. Rotation time was 0.25 s. CTDIvol was matched to that of the EID‐CT scans. The detector collimation was 144 × 0.4 mm. Virtual monochromatic images at 50, 60, and 70 keV were reconstructed with a slice thickness of 1.5 mm and an increment of 1.0 mm, using both Br44‐3 and Qr44‐3 kernels.

**FIGURE 2:**
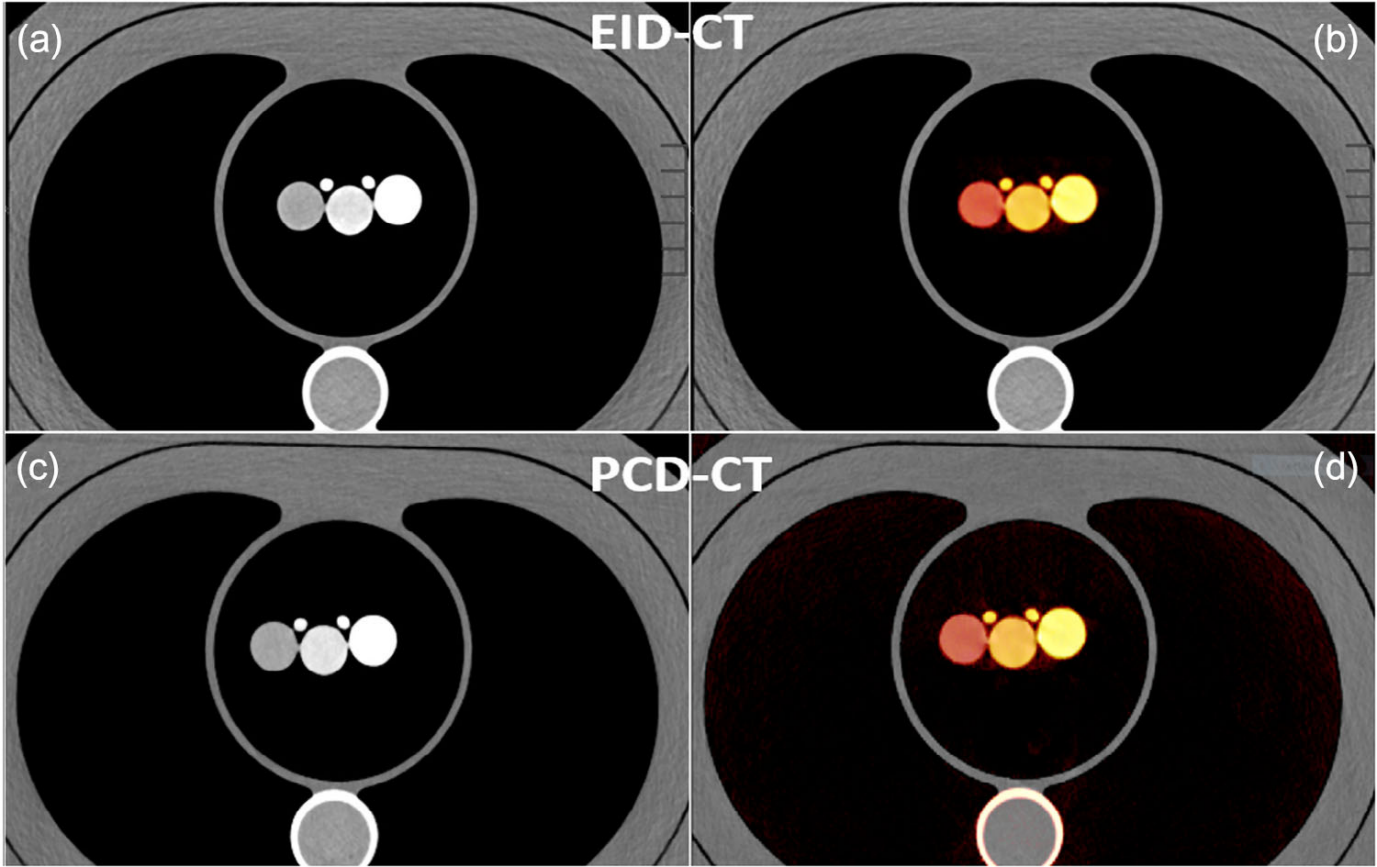
Grayscale CT image of the phantom with generated iodine color maps after multi‐energy processing for EID‐CT (a and b) and PCD‐CT (c and d). EID, energy‐integrating detector; PCD, photon‐counting‐detector.

From the ME scans on EID‐CT and PCD‐CT, iodine maps were generated using the lung analysis application of the *Syngo.via* workstation (Siemens Healthineers) (Figure 2a–d). This approach was based on a three‐material decomposition algorithm, allowing the generation of a virtually unenhanced image series and an iodine map image volume. Iodine concentrations were measured by placing circular ROI (area 1.2 cm^2^) in the center of each sample tube. The same ROI was propagated through five slices, and the average CT value (HU ± SD) and iodine concentration (mg/mL) were recorded. Root‐mean‐squared error (RMSE) and standard deviation (SD) were calculated for motion measurements for both scanners. RMSE and SD were used then as a measure of accuracy compared to ground truth data.

The percentage of simulated thrombus occlusion of the vessel was calculated on images without a motion as a baseline and compared with the percent thrombus occlusion from the images with 20 and 30 rpm motion, assuming that each tube represents the complete segment of the vessel. The area of interest was manually selected and adjusted on 3D images to ensure proper boundary selection, and the percentage of thrombus occlusions were estimated. For volume calculation, images reconstructed with Br44‐3 and Qr44‐3 were used to ensure accurate measurements as possible. Dice coefficient (D) between two sets was used as a measure of similarity hence accuracy. Calculated D for thrombus in tube 1 was 0.996 and 0.998 for thrombus in tube 2. For percent thrombus occlusion, measurements were based on 3D reconstructed images using the volume calculator, ImageJ (National Institutes of Health, Bethesda, Maryland, USA, https://imagej.nih.gov/ij/, 1997−2018*)*.

All statistical analysis was performed using IBM SPSS Statistics for Windows OS, Version 28.0.1.1 (IBM Corp., Armonk, New York, USA).

## 3 RESULTS

**FIGURE 3:**
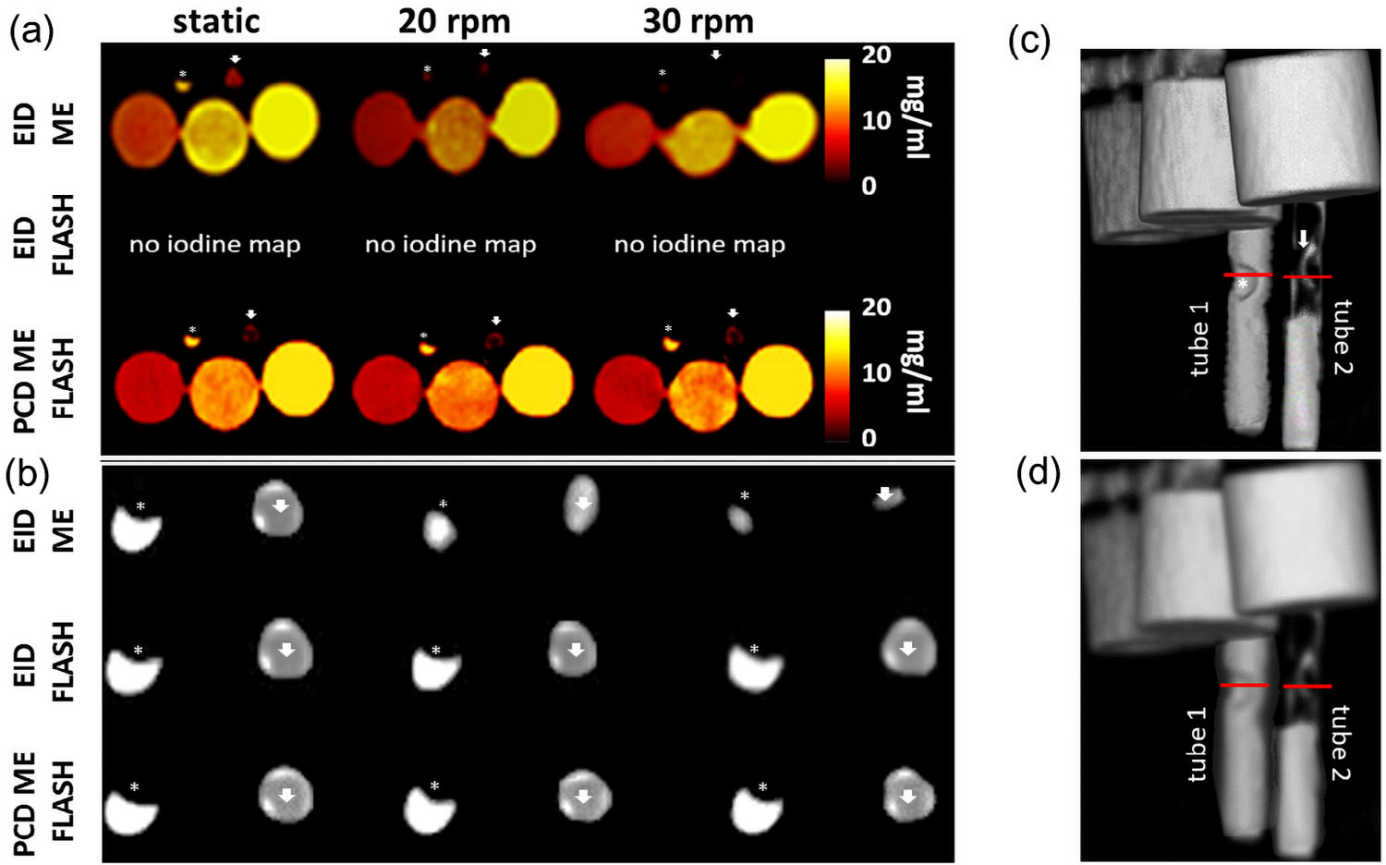
Influence of temporal resolution on iodine quantification and vessel visualization. (a) The first three rows show the iodine images of three iodine samples along with vessels acquired from EID ME (top row), EID high‐pitch (middle row), and PCD ME high‐pitch (bottom row) at stationary, 20, and 30 rpm, respectively. Note that EID high‐pitch cannot generate iodine maps. (b) The last three rows show the enlarged images of two PE mimicking tubes (white star showing emboli in tube 1 and white arrow points to the emboli in tube 2) acquired from EID ME, EID high‐pitch and PCD ME high‐pitch at rest, 20, and 30 rpm, respectively, through the slice indicated with the red line on image (c) and (d). (c) 3D image of the phantom on stationary images acquired with EID‐CT high‐pitch and with 20 rpm acquired with EID‐CT ME (d). EID, energy‐integrating detector; ME, multi‐energy; PCD, photon‐counting‐detector; PE, pulmonary embolism.

On the images without motion, both EID‐CT (high‐pitch and ME) and PCD‐CT maintain the circular shapes of the iodine tubes with high detectability without substantial distortion of the pulmonary emboli (Figure 3a). Figure 3b is a zoom‐in display of the two tubes mimicking PE in vessels on the greyscale image through representative slice indicated with red line in Figure 3c and d. The motion with 20 and 30 rpm lowered the visualization of PE when acquired at ME mode with EID‐CT (Figure 3b, top row) with apparent distortion in the tube shape compared to PCD‐CT high‐pitch ME (Figure 3a and b, bottom rows), where the shape of the tubes and PE was preserved and looked similar as on the image without motion. In contrast, motion artifact reduction was evident in images acquired at both EID‐CT high‐pitch mode and PCD‐CT (Figure 3b, middle row) due to the improved temporal resolution. Although the EID‐CT high‐pitch mode has a high temporal resolution matching the one on PCD‐CT, it cannot provide spectral information for iodine quantification. Measured iodine concentrations are summarized in Table 1.

**TABLE 1:** Measured iodine concentrations on EID‐CT and PCD‐CT without motion (stationary) and with motion (20 and 30 rpm).

	5 mg/mL		10 mg/mL		15 mg/mL	
	EID‐CT mean ± SD	PCD‐CT mean ± SD	EID‐CT mean ± SD	PCD‐CT mean ± SD	EID‐CT mean ± SD	PCD‐CT mean ± SD
Stationary	5.40 ± 0.27	5.10 ± 0.25	10.30 ± 0.52	10.60 ± 0.53	15.60 ± 0.80	15.30 ± 0.30
20 rpm	4.70 ± 0.24	5.20 ± 0.36	9.40 ± 0.90	10.90 ± 0.60	16.20 ± 0.70	15.90 ± 0.95
30 rpm	6.50 ± 0.70	5.20 ± 0.10	10.30 ± 1.10	10.70 ± 0.54	14.40 ± 1.15	15.90 ± 0.90
Repeated scans in motion						
20 rpm	4.70 ± 0.25	5.20 ± 0.26	9.80 ± 0.91	10.50 ± 0.55	16.60 ± 0.86	15.80 ± 0.73
30 rpm	4.20 ± 0.34	5.40 ± 0.31	10.40 ± 0.36	10.80 ± 1.00	15.40 ± 0.21	16.10 ± 0.69

Abbreviations: EID, energy‐integrating detector; PCD, photon‐counting‐detector.

**FIGURE 4:**
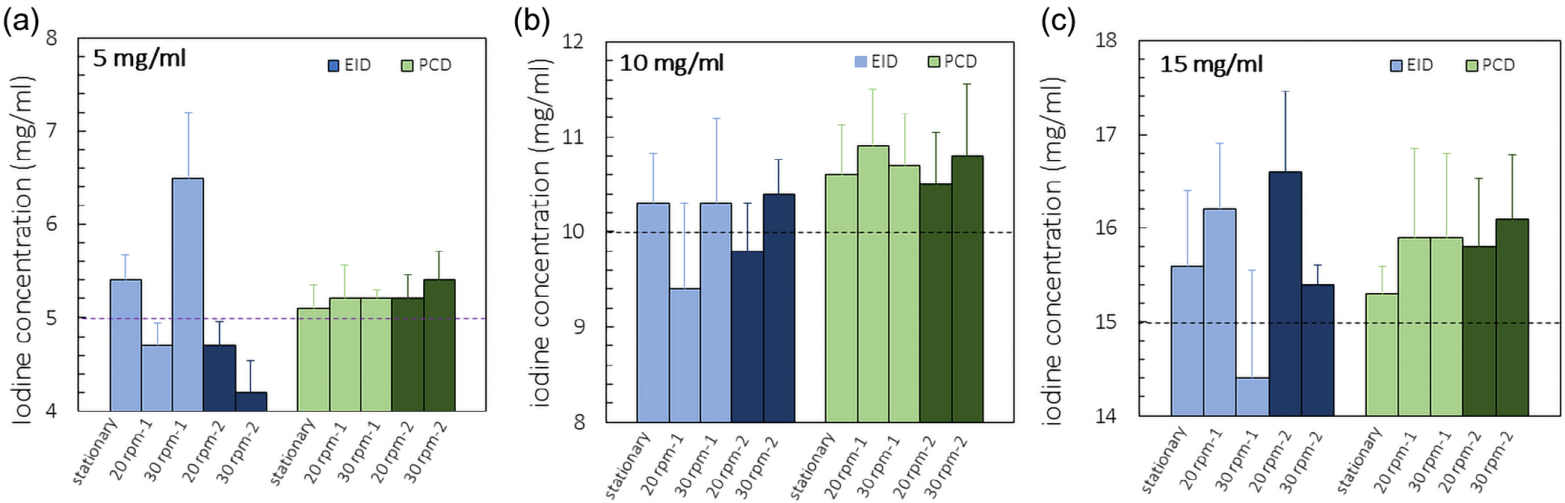
Measured iodine concentration in tubes containing 5 mg/mL (a), 10 mg/mL (b), and 15 mg/mL (c). Darker shades on the graph represents repeated scans labeled as 20 rpm‐2 and 30 rpm‐2. Black dashed lines on the graphs are the true iodine concentrations.

There was no significant difference (*p* > 0.05) between measured iodine concentrations between the two scanners when there was no motion (Figure 4a–c). Measured iodine concentrations from static images strongly correlated with the ground truth for EID‐CT at ME and PCD‐CT high‐pitch ME mode for three iodine tubes. Measured concentrations started to deviate more from the actual value when there was motion, with more differences from the static condition for EID‐ than PCD‐CT. Repeated scans with 20 and 30 rpm showed a similar trend with greater instability in measured iodine concentrations using EID‐CT. At the same time, less variation was observed between ground truth and the first and repeated measurements for PCD‐CT (Figure 4a–c). Calculated RMSE and SD at motion are summarized in Table 2. Lower SD values for PCD‐CT in high‐pitch ME mode were evident compared to EID‐CT in ME mode.

**TABLE 2:** Root‐mean‐squared error (RMSE) and standard deviation (SD) for multi‐energy EID‐CT and multi‐energy high‐pitch PCT‐CT calculated for measurements at motion averaged over both speeds.

	5 mg/mL		10 mg/mL		15 mg/mL	
	EID‐CT	PCD‐CT	EID‐CT	PCD‐CT	EID‐CT	PCD‐CT
RMSE	0.30	0.13	0.19	0.36	0.49	0.46
SD	0.88	0.09	0.40	0.15	0.84	0.11

Abbreviations: EID, energy‐integrating detector; PCD, photon‐counting‐detector.

The visual detection and calculated percentage of PE were similar when EID‐CT at high‐pitch and ME and PCD‐CT at high‐pitch ME mode were compared in the static images. As expected, the accuracy of the measurements dropped significantly for EID‐CT ME mode with applied motion. All results are summarized in Table 3.

**TABLE 3:** The percentage of the thrombus occlusion calculated for different CT systems and acquisition modes.

Percent thrombus occlusion (%)	EID‐CT ME		EID‐CT High‐pitch		PCD‐CT High‐pitch ME	
	tube 1	tube 2	tube 1	tube 2	tube 1	tube 2
static	53.1 ± 1.3	85.7 ± 2.1	54.2 ± 1.3	83.1 ± 2.0	55.6 ± 1.3	82.2 ± 2.0
20 rpm	79.5 ± 4.0	91.9 ± 4.6	53.9 ± 2.7	81.8 ± 4.1	55.9 ± 2.8	83.0 ± 4.2
30 rpm	80.1 ± 6.4	91.7 ± 7.3	56.4 ± 4.5	84.1 ± 6.7	56.7 ± 4.5	84.4 ± 6.7

Abbreviations: EID, energy‐integrating detector; ME, multi‐energy; PCD, photon‐counting‐detector.

**FIGURE 5:**
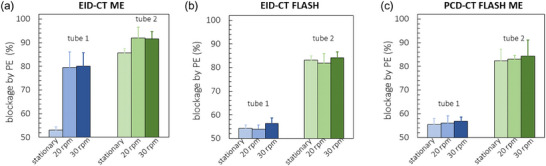
Comparison of the blockage percentage for EID‐CT ME (a), EID‐CT high‐pitch (b), and PCD‐CT high‐pitch ME (c) acquisition modes without and with a motion. EID, energy‐integrating detector; ME, multi‐energy; PCD, photon‐counting‐detector.

In tube 1, the calculated blockage was 53.1 ± 1.3%, while in tube 2, it was 85.7 ± 2.1% for EID‐CT ME and 55.6 ± 1.3% and 82.2 ± 2.0% for PCD‐CT high‐pitch ME on static images. In contrast, on images with 20 and 30 rpm, the calculated percentage of thrombus occlusion in tube 1 was estimated to be 79.5 ± 4.0% and 80.1 ± 6.4% for EID‐CT ME and 55.9 ± 2.8% and 56.7 ± 4.5% for PCD‐CT high pitch ME on images with 20 and 30 rpm, respectively. Values for EID‐CT ME at motion are much higher than those calculated on static images. EID‐CT high‐pitch mode (Figure 5b) agreed well with PCD‐CT high‐pitch ME (Figure 5c) on the static and images with 20 and 30 rpm. Values in tube 2 agreed well with static images for both scanners. RMSE and SD supports these findings. Higher values for multi‐energy EID‐CT at motion averaged over both speeds, especially for tube 1 were observed (Table 4).

**TABLE 4:** Root‐mean‐squared error (RMSE) and standard deviation (SD) for multi‐energy EID‐CT and multi‐energy (ME) high‐pitch PCD‐CT calculated for measurements at motion averaged over both speeds for the estimated percentage of thrombus occlusion.

	EID‐CT ME		EID‐CT High‐pitch		PCD‐CT High‐pitch ME	
	tube 1	tube 2	tube 1	tube 2	tube 1	tube 2
RMSE	18.8	4.3	0.7	0.1	0.6	1.1
SD	0.3	0.1	1.2	1.1	0.4	0.7

Abbreviations: EID, energy‐integrating detector; PCD, photon‐counting‐detector.

## 4 DISCUSSION

This phantom study evaluated the benefits of a simultaneous high‐pitch and ME acquisition mode using a PCD‐CT system in imaging PE. Compared to the ME mode in EID‐CT, the high‐pitch imaging in PCD‐CT reduced motion artifacts and generated higher accuracy in iodine quantification. Compared to the high‐pitch mode in EID‐CT, PCD‐CT acquisition had inherent ME imaging capability, which makes iodine quantification and other spectral imaging possible.

The iodine and PE tube visualization due to motion artifact was significantly decreased due to the slower temporal resolution at EID‐CT ME mode compared to EID high‐pitch mode.^13^ The PCD‐CT high‐pitch ME mode could “stop” the motion because of its superior temporal resolution, preserving the shape and size of the tubes and simultaneously allowing the generation of iodine maps.

We found that with PCD‐CT, measured values for iodine density were slightly higher than actual, with good reproducibility on repeated scans even with the motion. Measured concentrations were not reproducible with EID‐CT. On repeated scans, concentrations were higher/lower than the actual values. The reason for better performance of PCD‐CT could be how dual energy mode works. The two tubes operate at different kVs, and half‐rotation is needed from each tube, lowering the temporal resolution and preventing perfect alignment of moving structures. Namely, the orthogonal offset between the two x‐ray tubes in dual‐source CT is ∼95°. In the dual‐energy mode, these x‐ray tubes are operated at two different kVs, one at low and the other at high energy. The offset of low‐ and high‐energy projection data does not allow projection‐domain spectral reconstruction. Image‐domain spectral reconstruction is associated with higher beam hardening and imperfect material separation, causing the misregistration artifact for moving or high contrast structures.^23^, ^24^ This effect depends highly on the type of dual‐energy CT scanner and the scan parameters used.^24^, ^25^ This offset between two sources can introduce different patterns of motion artifacts and spatial misregistration, which could explain the variations in measured concentrations.

However, higher temporal resolution achieved with PCD‐CT is expected to generate more stable iodine measurements. A lower standard deviation was calculated for PCD‐CT high‐pitch ME measurements at motion, indicating data clustered tightly around the mean compared to EID‐CT, where SD had higher values and data were more spread out. Slightly higher measured concentrations than ground truth were seen for 10 and 15 mg/mL tubes for both scanners, possibly due to the calibration differences.

Quantifying the extent of the percent thrombus occlusion from the images with a motion showed a significant decrease in measurements when EID‐CT ME mode was used. Images with 30 rpm introduced vast distortion of the shape of the phantom. In these images, estimating the extent of PE was challenging. Under these conditions, the percent thrombus occlusion appeared to be higher due to the motion‐induced distortion.

One of the limitations of this study is that we only repeated the scans twice for each motion condition. Motion artifact may depend on the relative positions of the x‐ray tubes and the motion of the objects, so repeating the scans more than two times would be better for assessing the variability in motion artifact. However, as shown in Figure 4 and Table 2, because of the much‐reduced motion artifacts in PCD high‐pitch scans, the iodine concentration measurements had much more stable results compared to those from the EID ME mode, which suffered from variations of motion artifacts among different scans. To a large extent, this already demonstrated the benefits of high temporal resolution on PCD high‐pitch mode: with much reduced motion artifacts, the measurement was more stable. In addition, it should also be noted that CT scanners are busy clinical scanners with limited availability, which together with relatively complex experimental setup limits possibility for longer scans.

Another limitation of our study is that this was a phantom study without anatomical movement artifacts from breathing and pulsation. To fully assess the potential benefits, the results from this study should be leveraged in a clinical setting.

## 5 CONCLUSION

The combined ME and high‐pitch acquisition mode in dual‐source PCD‐CT enables simultaneous accurate iodine quantification and high‐pitch motion‐free PE imaging.

## AUTHOR CONTRIBUTIONS

Jelena M. Mihailovic: data curation (equal), visualization (lead), formal analysis (lead), methodology (equal), writing‐original draft (lead), writing‐review and editing (equal); Michael R. Bruesewitz: investigation (equal), methodology (equal); Joseph R. Swicklik: investigation (equal), methodology (equal); Mariana Yalon: writing‐review and editing (equal); Prabhakar Rajiah: conceptualization (supporting), writing‐review and editing (equal); Joel G. Fletcher: conceptualization (equal), investigation (equal), methodology (equal), funding‐acquisition (lead), writing‐reviewing and editing (equal); Cynthia H. McCollough: conceptualization (equal), investigation (equal), methodology (equal), funding acquisition (lead), writing‐reviewing and editing (equal); Lifeng Yu: conceptualization (lead), formal analysis (equal), visualization (equal), investigation (equal), methodology (equal), supervision (lead), writing‐reviewing and editing (equal)

Portions of this paper were presented at RSNA 2023.

## CONFLICT OF INTEREST STATEMENT

Cynthia H. McCollough and Joel G. Fletcher are the recipients of a research grant to their institution from Siemens Healthineers. The remaining authors report nothing to disclose.

## Data Availability

The data that support the findings of this study are available from the corresponding author upon reasonable request.
